# Analysis of high-risk factors for preoperative DVT in elderly patients with simple hip fractures and construction of a nomogram prediction model

**DOI:** 10.1186/s12891-022-05377-8

**Published:** 2022-05-11

**Authors:** Liang Zhang, Miao He, Wenlong Jia, Wenqing Xie, Ya Song, Haochen Wang, Jiangnan Peng, Yusheng Li, Zhaohui Wang, Zhangyuan Lin

**Affiliations:** 1 Departmrnt of Orthopaedic Trauma, The Affiliated Chenzhou Hospital, Hengyang Medical School, University of South China, Hengyang, 423000 Hunan China; 2 Department of Orthopaedics, Xiangya Hospital, Central South University, Changsha, 410008 Hunan China; 3 Department of Orthopaedics, The First Affiliated Hospital of Zhengzhou University, Zhengzhou, 450052 Henan China

**Keywords:** Hip fracture, DVT, Risk factors, ROC, Nomogram prediction model

## Abstract

**Background:**

Hip fractures are anatomically classified in relation to femoral neck, intertrochanteric or subtrochanteric fractures. Simple hip fractures discussed in this study are femoral neck fractures or intertrochanteric fractures, which are the most common types of hip fractures. Controversy remains regarding the value of biochemical indices of thrombosis in elderly patients with fractures. A retrospective study was conducted to investigate the index admission data in blood draws of elderly patients with hip fractures and their high-risk factors for deep venous thrombosis (DVT). A nomogram prediction model for DVT was established to facilitate a rapid, accurate, and effective prediction based on the results.

**Methods:**

The data were based on 562 elderly patients undergoing hip fracture surgery, from whom 274 patients were selected for enrollment. The 274 patients were divided into two groups using preoperative vascular color Doppler ultrasonography. Chi-square tests, t-tests, and U tests were conducted, and logistic regression analysis was conducted showing different factors between the two groups. Independent risk factors with statistical significance (*P* < 0.05) were obtained, and the logistic regression equation and the new variable prediction probability_1 (PRE_1) were constructed. The receiver operating characteristic (ROC) curve of risk factors and PRE_1 was drawn to obtain the area under the curve (AUC) and truncation value of each risk factor. Finally, a nomogram prediction model was constructed using the R programming language to calculate the concordance index (C-index).

**Results:**

Time from injury to hospitalization, platelet (PLT) count, D-dimer level, fibrinogen (FIB) level, and systemic immune-inflammatory index (SII) score were independent risk factors for preoperative DVT in elderly patients with hip fractures. The logistic regression equation and PRE_1 were constructed by combining the above factors. ROC analysis showed that the area under the curve for PRE_1 (AUC = 0.808) was greater than that of the other factors. The sensitivity of PRE_1 (sensitivity = 0.756) was also higher than that of the other factors, and the specificity of PRE_1 (specificity = 0.756) was higher than that of two other factors. Moreover, a predictive nomogram was established, and the results showed a high consistency between the actual probability and the predicted probability (C-index = 0.808), indicating a high predictive value in fractures accompanied by DVT.

**Conclusions:**

This study confirmed that SII score could be used as a risk factor in the prediction of DVT occurrence. A nomogram prediction model was constructed by combining 5 independent risk factors: time from injury to admission, PLT count, D-dimer level, FIB level, and SII score, which had high predictive values for fractures accompanied by DVT. This model use is limited to simple hip fracture.

## Background

Hip fractures are one of the most common types of fractures in the elderly population. In 1990, the number of hip fractures was 1.66 million per year worldwide and was predicted to be 6.26 million annually by the mid-2000s [1]. Meanwhile, deep venous thrombosis (DVT) is the most dangerous complication of hip fracture. Medical studies have generally shown that DVT is a common condition that induces morbidity of other diseases, pulmonary embolism (PE) and even death in patients with fracture trauma [2]. Currently, DVT is often monitored by some indices that clinically reflect the coagulation status, such as D-dimer level, fibrinogen (FIB) level, and platelet count (PLT). A high serum level of D-dimer suggests a hypercoagulable and hyperfibrinolytic state, so D-dimer level has a suggestive effect in patients with suspected DVT [3]. However, controversy remains regarding the value of D-dimer in elderly patients with fractures [4]. FIB promotes blood coagulation in the final stage of coagulation. D-dimer and FIB are both clinically used biochemical indices of thrombosis. However, D-dimer and FIB can also increase in nonfracture patients, in which case the observation of a single index has the characteristics of a low specificity and high false-positive rate in the diagnosis of DVT [5]. In addition, the increase in PLT count also suggests a hypercoagulable state [6]. In fact, preoperative DVT in elderly patients with hip fracture is related to time from injury to admission. This study [7, 8] showed that the incidence of DVT rises approximately 54.5% ~ 62% when the admission time of hip fracture patients is delayed for more than 48 hours. According to the Chinese guidelines for the Prevention of Venous Thromboembolism in Orthopedic surgery [9], color Doppler ultrasonography with high sensitivity and accuracy has gradually become the preferred auxiliary examination to confirm DVT by replacing vein photography. However, with a color Doppler ultrasound, it is difficult to show a clear image of the patient’s hip fracture in a fixed body position, serious limb swelling, severe pain and so on.

Recently, many studies have shown that the immune inflammatory system plays a key role in promoting the formation of DVT [10, 11]. A potential mechanism is shown by these findings: the formation of DVT is caused by the cascade reaction of inflammatory cytokines and chemokines. The immune inflammatory status can be reflected by many peripheral blood-derived indices, including the monocyte/lymphocyte ratio (MLR), neutrophil/lymphocyte ratio (NLR), platelet/lymphocyte ratio (PLR) and systemic immune inflammatory index (SII, SII=PLT × NC/LC) [12, 13]. These indices have been proven to be closely related to inflammation and immune status and have good predictive value for the prognosis of various infectious, neoplastic and autoimmune diseases. Moreover, these peripheral blood-derived indices have been increasingly applied in major orthopedic surgeries [14–16]. Compared with the MLR, the NLR and PLR can comprehensively reflect the balance among inflammation, blood coagulation and immune state as a novel index of systemic immune inflammation. These various combinations of peripheral blood-derived indices are increasingly used in studies of fracture complications [17]. Currently, there is a lack of a reliable predictive model to synthesize all risk factors, so a novel way to construct a predictive model combined with known risk factors to predict DVT is attractive, and a simpler and more effective auxiliary examination is urgently needed to evaluate the existence of DVT in hospitalized patients.

## Methods

Research object: Elderly patients who planned to undergo hip fracture surgery from January 2015 to December 2020 in the Orthopedic Department of Xiangya Hospital affiliated with Central South University. A total of 562 elderly patients with hip fracture were enrolled (age ≥ 60 years), including 117 patients with multiple fractures (fracture site≥2), 1 patient with pathological fracture, 2 patients with a previous history of thrombosis, 29 patients with previous and recent use of anticoagulant drugs, 38 patients with inflammatory diseases, and 13 patients with uncertain admission diagnosis. Twelve patients had coagulation disorders, 34 patients had incomplete examination data, and 42 patients had an injury-admission time ≥ 1 week. Finally, 274 patients were enrolled.

**Fig. 1:**
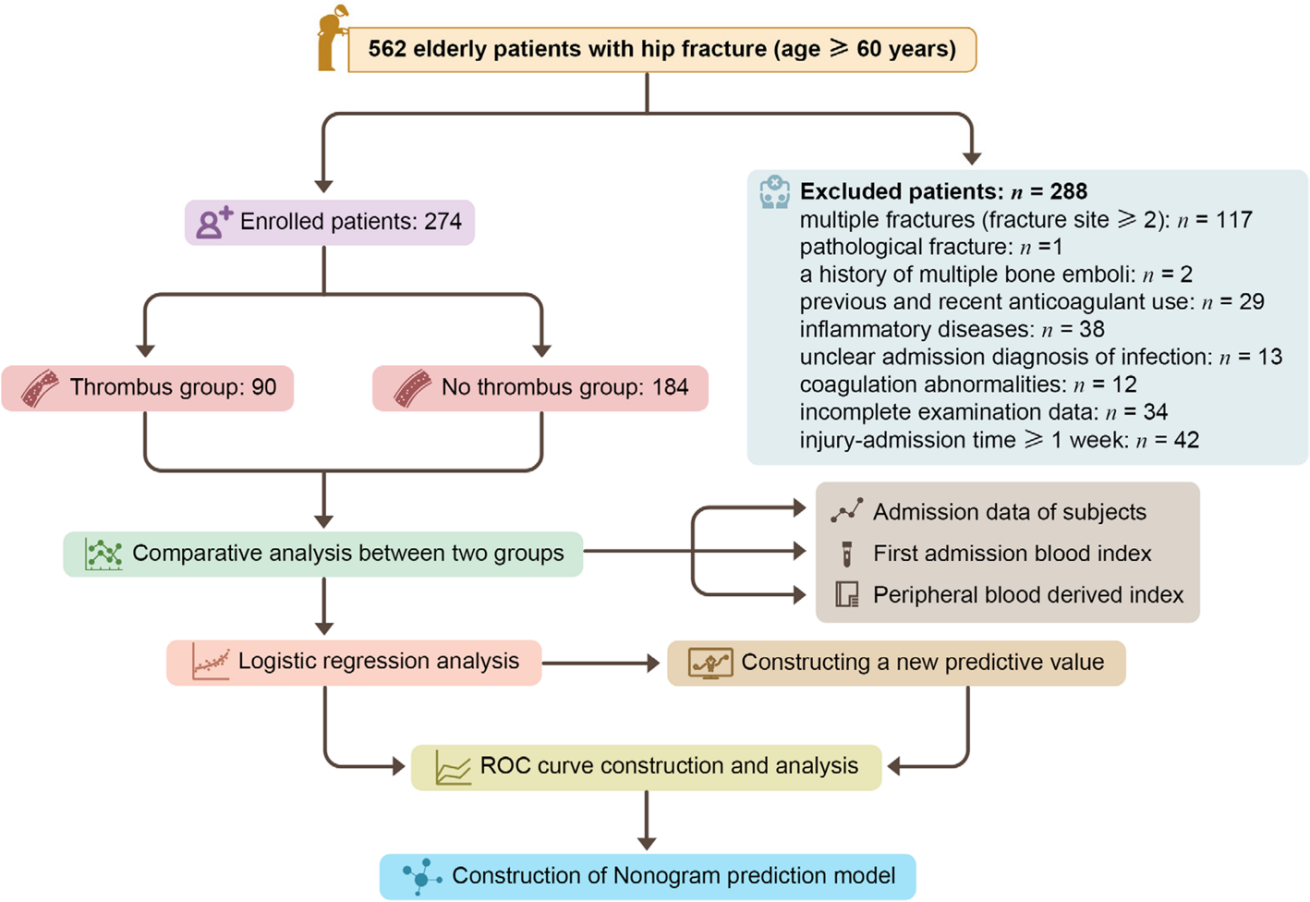
Collection procedure of all elderly patients with hip fracture in this study

The blood vessels of the lower extremities screened were bilateral veins, including the common femoral vein, superficial femoral vein, deep femoral vein, popliteal vein, anterior tibial vein, posterior tibial vein and peroneal vein. Among these, distal DVT included a thrombus of the anterior tibial vein, posterior tibial vein, peroneal vein and their combination; proximal DVT included a thrombus of the popliteal vein and proximal popliteal vein; and mixed DVT included the simultaneous presence of thrombi in the proximal and distal deep veins. Superficial vein thrombi were not involved in this study due to the low clinical importance of superficial veins (great saphenous veins or small saphenous veins) or myenteric veins (such as soleus veins or gastrocnemius veins). Color Doppler ultrasound was used by experienced sonographers to examine the blood vessels of the lower extremities to determine the existence of DVT. The diagnostic criteria of DVT by color Doppler ultrasound mainly included the following: 1. Abnormal echoes. 2. At the injured site, the vein under the ultrasound probe should not be closed by pressing. 3. No obvious blood flow signal in the venous thrombus segment. 4. Decrease in the vascular width and blood flow of the affected limb. Patients were divided into two groups using preoperative vascular color Doppler ultrasonography: those with DVT (thrombus group; 90 cases) and those without DVT (nonthrombus group; 184 cases), and the grouping criteria are shown in the flow chart (Fig. 1).

Statistical analysis: The database including the admission data and blood draws of the two groups was established by Excel, and the statistical analysis was performed by SPSS 22 software. The Shapiro–Wilk test and Levene test were used to determine the normal distribution and homoscedasticity, respectively. Then, the measurement data were described as the mean ± standard deviation (mean ± SD); otherwise, the measurement data were expressed as the median [M(range)], and the numerical data were expressed as a case (percentage)[N(%)]. Through screening, the chi-square test, t-test and Mann–Whitney U test were used to compare the basic admission data. Hematology index scores and peripheral blood derivative index scores among 274 elderly patients with hip fracture were calculated on admission, and the statistically significant factors between the two groups are listed (*P* < 0.05). The risk factors (*P* < 0.05) were further obtained by logistic regression analysis of the statistically significant factors after testing. According to the logistic regression coefficient, the logistic regression equation and the new variable prediction probability_1 (PRE_1) were constructed. The risk factors in the equation and PRE_1 were used to draw the receiver operating characteristic (ROC) curve, and the area under the curve (AUC), and cutoff values for each risk factor were obtained. The independent risk factors identified by logistic regression analysis were processed in R to further construct a nomogram prediction model and calculate the consistency index (c-index).

## Results

The data of the 274 patients were divided into two groups using preoperative vascular color Doppler ultrasonography: those with DVT (thrombus group) and those without DVT (nonthrombus group). The thrombus group (*N* = 90,33%), in which 66 patients had swelling or pain, was divided further into three groups: proximal thrombus (*N* = 34, 37.8%), distal thrombus (*N* = 29, 32.2%) and mixed thrombus (*N* = 27, 30%), according to thrombus location. There were 58 males (64.4%) and 32 females (35.6%) in the thrombus group, whose average age and BMI were 68.3 ± 6.6 years and 22.6 ± 1.7 kg/m2. There were 124 males (67.4%) and 60 females (32.6%) in the nonthrombus group, whose average age and BMI were 68.5 ± 6.1 years and 22.4 ± 1.9 kg/m2. Among the patients with hip fracture, there were 52 (57.8%) femoral neck fractures and 38 (42.2%) intertrochanteric fractures in the thrombus group according to fracture site, compared to the nonthrombus group, in which 103 (56.0%) had femoral neck fractures and 81 (44.0%) had intertrochanteric fractures. Considering the injury mechanism, there were 76 patients (84.4%) with low-energy injuries in the thrombus group compared to 159 patients (86.4%) in the nonthrombus group and 14 patients (15.6%) with high-energy injuries in the thrombus group compared to 25 patients (15.6%) in the nonthrombus group. There was no significant difference in personal history, previous history or the above indices between the thrombus group and the nonthrombus group (*P* > 0.05). There was a significant difference in the time from injury to hospitalization between the thrombus group (3.08 ± 1.37 d) and the nonthrombus group (2.2 ± 1.06 d) (*P* < 0.001). The Caprini thrombus risk score was 8.97 ± 1.00 points in the thrombus group and 8.72 ± 1.24 points in the nonthrombus group, which did not show a significant difference between the two groups (*P* > 0.05) (Table 1).

**Table 1:** Basic information in the Admission of elderly patients with hip fracture

Basic information of admission	Thrombus group (*N* = 90)	Non-thrombus group (*N* = 184)	χ^2^/t/Z	*P*
Gender (name)				
Male	58(64.4%)	124(67.4%)	0.24	0.63
Female	32(35.6%)	60(32.6%)		
Age (years)	68.3 ± 6.6	68.5 ± 6.1	−0.19	0.85
Fracture site				
Femoral neck fracture	52(57.8%)	103(56.0%)	0.08	0.78
Intertrochanteric (inferior) fracture	38(42.2%)	81(44.0%)		
BMI(Kg/m^2^)	22.6 ± 1.7	22.4 ± 1.9	1.23	0.35
Damage mechanism				
Low energy damage	76(84.4%)	159(86.4%)	0.19	0.66
High energy damage	14(15.6%)	25(13.6%)		
Smoking history				
Yes	15(16.7%)	18(9.8%)	2.70	0.10
No	75(83.3%)	166(90.2%)		
Drinking history				
Yes	23(25.6%)	55(29.9%)	0.56	0.42
No	67(74.4%)	129(70.1%)		
Previous history				
Hypertension				
Yes	37(41.1%)	56(30.4%)	3.07	0.08
No	53(58.9%)	128(69.6%)		
Diabetes				
Yes	26(28.9%)	57(31.0%)	0.13	0.72
No	64(71.1%)	127(69.0%)		
Coronary disease				
Yes	12(13.3%)	19(10.3%)	0.55	0.46
No	78(86.7%)	165(89.7%)		
Injury - admission time (days)	3.08 ± 1.37	2.2 ± 1.06	5.35	<0.001
Caprini thrombus risk score	8.97 ± 1.00	8.72 ± 1.24	1.79	0.75

After admission, a set of blood sample tests were performed to complete the study data, such as RBC (WBC, PLT, NC, MC, LC), routine coagulation (PT, APTT, TT, D-dimer, FIB), ESR, and CRP. There were significant differences in WBC, PLT, NC, MC, D-dimer, CRP and FIB levels between the thrombus group and the nonthrombus group (*P* < 0.05), while LC, PT, APTT, and TT levels were not statistically significant (*P* > 0.05) (Table 2). Peripheral blood-derived indices such as the MLR, NLR, PLR, and SII were obtained from a combination of various indices of routine blood tests; among these, there was a significant difference in the MLR and NLR between the thrombus group and the nonthrombus group (*P* < 0.05). In addition, the levels of PLR and SII in the thrombus group were significantly higher, and the differences were statistically significant compared with the patients without thrombosis (*P* < 0.001) (Table 3).

**Table 2:** Admission data using first hematological index results of elderly patients with hip fracture

Hematological index	Thrombus group(*N* = 90)	Non-thrombus group (*N* = 184)	χ^2^/*t*/Z	*P*
WBC	7.9(6.5 ~ 9.2)	7.0(5.9 ~ 8.6)	−2.4	0.015
PLT	176(136 ~ 235)	142(118 ~ 185)	−3.8	<0.001
NC	5.8(4.9 ~ 7.3)	5.1(4.1 ~ 6.6)	−2.9	0.003
MC	0.7(0.5 ~ 0.9)	0.6(0.5 ~ 0.8)	−2.1	0.039
LC	1.0(0.7 ~ 1.4)	1.1(0.7 ~ 1.4)	−1.1	0.281
PT	13.75 (13.08 ~ 14.43)	13.40 (12.80 ~ 14.30)	−1.5	0.120
APTT	31.65 (29.95 ~ 34.76)	32.60 (28.90 ~ 35.30)	−0.3	0.710
TT	16.45 (15.85 ~ 18.13)	16.50 (15.40 ~ 17.90)	−0.4	0.660
D-dimer	1.12(0.65 ~ 1.52)	0.78(0.45 ~ 1.29)	−3.3	0.001
FIB	4.63 ± 1.22	4.01 ± 1.23	3.90	<0.001
CRP	56.20(35.98 ~ 85.23)	56.30(23.40 ~ 63.40)	−1.9	0.047
ESR	61.40(45.75 ~ 85.00)	62.40(39.00 ~ 66.00)	−1.4	0.136

*WBC* white blood cell count, *PLT* platelet count, *NC* neutrophil count, *MC* monocyte count, *LC* lymphocyte count, Partial indexes of coagulation routine: *PT* prothrombin time, *APTT* activated partial thromboplastin time, *TT* thrombin time, *FIB* fibrinogen, *CRP* C-reactive protein, *ESR* erythrocyte sedimentation rate

**Table 3:** Results of different peripheral blood derived indexes

Peripheral blood derived index	Thrombus group (*N* = 90)	Non-thrombus group (*N* = 184)	χ2/t/Z	*P*
MLR	0.70(0.50 ~ 1.00)	0.57(0.42 ~ 0.78)	−2.62	0.01
NLR	5.68(4.24 ~ 10.00)	5.00(3.60 ~ 7.25)	−2.43	0.02
PLR	177.7 (125.86 ~ 257.36)	144.51 (106.25 ~ 195.45)	−3.80	<0.001
SII	1012.1 (674.82 ~ 1786.25)	766.89 (544.71 ~ 1004.00)	−4.38	<0.001

MLR = MC/LC, NLR = NC/LC, PLR = PLT/LC, SII=PLT × NC/LC

Statistically significant factors using the time from injury to hospitalization, WBC, NC, MC, D-dimer, PLT, FIB, and CRP levels, and MLR, NLR, PLR, and SII (*P* < 0.05) were obtained by comparing between the two groups, and then the factors were analyzed by univariate logistic regression one by one. Univariate logistic regression analysis showed that there were significant differences in injury-admission time, WBC, NC, MC, D-dimer, PLT, and FIB levels, and PLR and SII, while there was no significant difference in CRP level, MLR or NLR (*P* > 0.05) (Table 4).

**Table 4:** Results of univariate Logistic regression analysis of preoperative DVT complication in elderly patients with hip fracture

Blood factor	*β*	S.E	Ward	OR	95% CI	*P*
Injury-admission time	0.584	0.113	26.618	1.794	1.437 ~ 2.240	<0.001
WBC	0.112	0.055	4.227	1.119	1.005 ~ 1.245	0.040
PLT	0.009	0.002	15.39	1.009	1.004 ~ 1.013	<0.001
NC	0.137	0.059	5.414	1.147	1.022 ~ 1.287	0.020
MC	0.855	0.470	3.305	2.350	0.935 ~ 5.906	0.069
D-dimer	0.642	0.214	9.01	1.899	1.249 ~ 2.888	0.003
FIB	0.401	0.108	13.787	1.493	1.208 ~ 1.845	<0.001
CRP	0.003	0.002	1.64	1.003	0.998 ~ 1.008	0.200
MLR	0.565	0.309	3.336	1.760	0.960 ~ 3.227	0.068
NLR	0.039	0.025	2.45	1.039	0.990 ~ 1.091	0.118
PLR	0.005	0.001	10.818	1.005	1.002 ~ 1.007	0.001
SII	0.001	0.000	15.713	1.001	1.000 ~ 1.001	<0.001

**Fig. 2:**
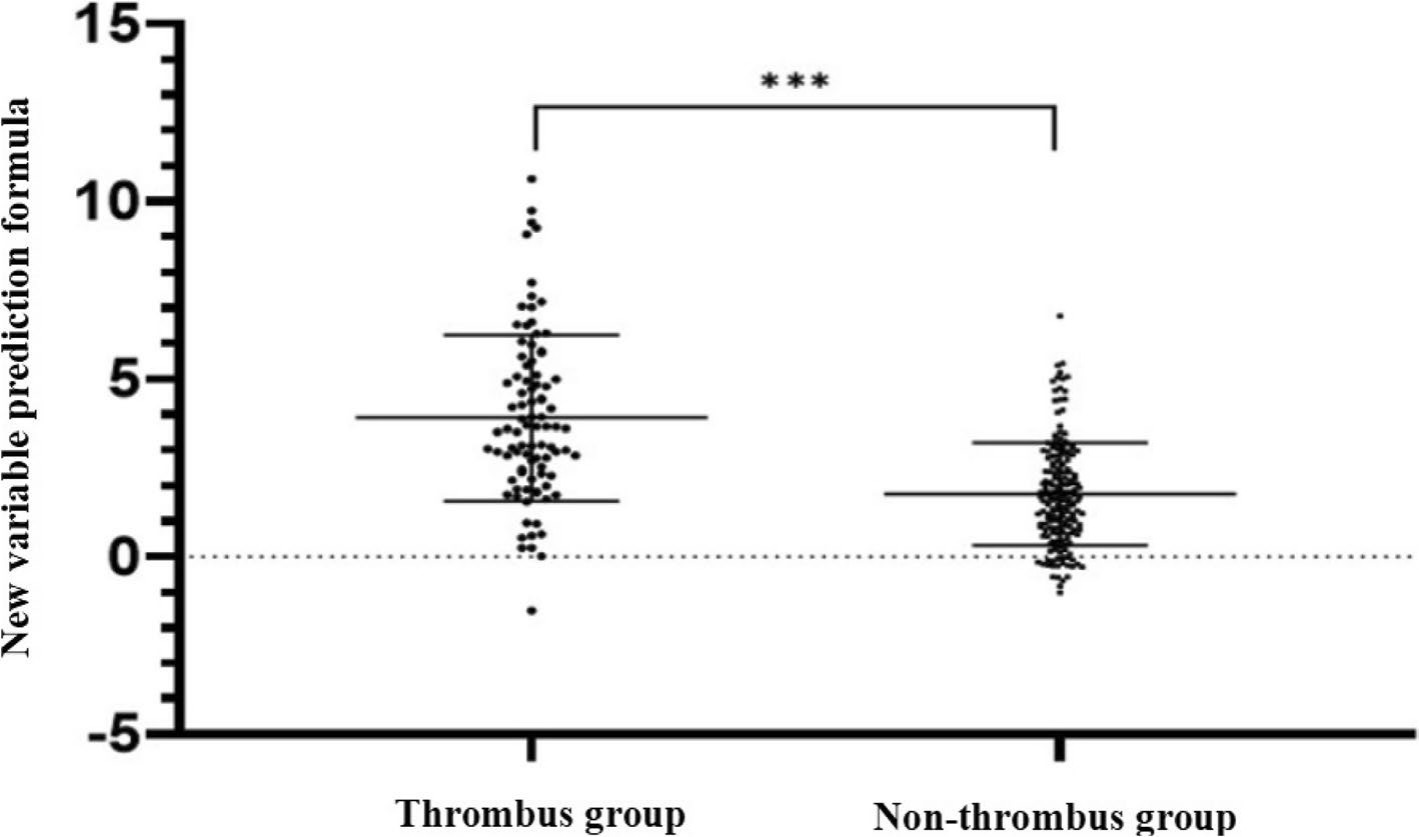
Scatter plot of the predicted values of the thrombosis group and the non-thrombosis group

The risk factors obtained by univariate logistic regression analysis were included in the multivariate logistic regression analysis to confirm that the five factors using the time from injury to hospital admission, D-dimer, PLT, and FIB levels and SII were independent risk factors affecting hip fracture complicated with DVT before surgery in elderly individuals (Table 5). The predictive formula (logit(p) = − 4.536 + 0.632 × injury-admission time + 0.008 × PLT + 0.698 × D-dimer + 0.33 × FIB+ 0.002 × SII) for new variables was constructed according to the regression coefficient (*β*) and the constant of risk factors. The results and the scatter plot (Fig. 2) were obtained by bringing the original data into the new variable prediction formula. The total sample (mean ± SD) was 2.5 ± 2.2, and there was a significant difference between the thrombus group (3.8 ± 2.3) and the nonthrombus group (1.8 ± 1.7) (*P* < 0.001).

**Table 5:** Results of multivariate Logistic regression analysis of preoperative DVT complication in elderly patients with hip fracture

Risk factor	*β*	S.E	Wald	OR	95% CI	*P*
Injury - admission time	0.632	0.126	25.284	1.881	1.470 ~ 2.405	<0.001
WBC	−0.217	0.220	0.965	0.805	0.523 ~ 1.241	0.326
PLT	0.008	0.003	7.603	1.008	1.002 ~ 1.015	0.006
NC	0.021	0.287	0.006	1.022	0.582 ~ 1.795	0.940
D-dimer	0.698	0.250	7.796	2.009	1.231 ~ 3.279	0.005
FIB	0.33	0.122	7.265	1.391	1.094 ~ 1.768	0.007
PLR	−0.01	0.006	2.769	0.990	0.978 ~ 1.002	0.096
SII	0.002	0.001	4.144	1.002	1.000 ~ 1.004	0.042
Constant	−4.536	1.331	11.613	~	~	~

**Fig. 3:**
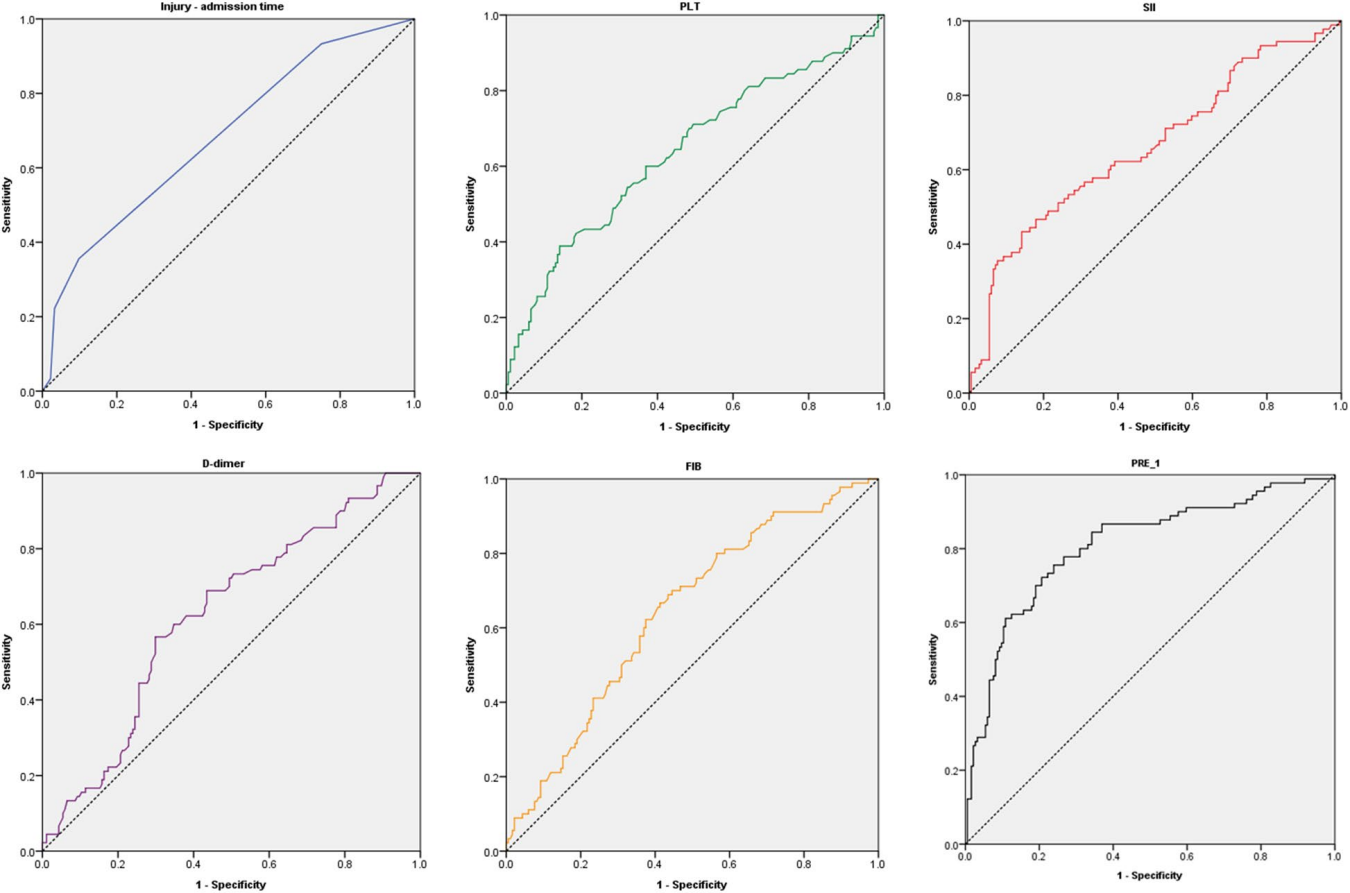
The ROC curve of PRE_1 and five independent risk factors

The ROC curve of PRE_1 and each independent risk factor was drawn (Fig. 3) by analyzing the above five independent risk factors and constructing PRE_1. The area under the curve (AUC) of PRE_1, the cutoff value, and the sensitivity and specificity were 0.808 (95% CI: 0.757 ~ 0.866), 0.707, 75.6 and 76.1%, respectively. The AUC values of injury to hospitalization time, PLT, D-dimer, FIB, and SII were 0.682, 0.642, 0.624, 0.641, and 0.663, respectively (*P* < 0.05), and the sensitivity and specificity are shown in Table 6. From this, the predictive efficiency of the model was high, while the sensitivity was better than all single independent risk factors, and the specificity was even better than two of the independent risk factors. Moreover, the Hosmer-Lemeshow goodness of fit test results of the predictive model (χ^2^ = 8.501, *P* = 0.386) (*P* > 0.05) indicated that the difference between the predicted value of the model and the actual observation value was not statistically significant, which suggests a good fit of the predicted model.

**Table 6:** ROC curve results of various risk factors and new predicted probability values

Prediction factor	Cut-off value	AUC	Sensitivity	Specificity	95% CI	*P*
Injury - admission time	3.5	0.682	0.356	0.902	0.615 ~ 0.750	<0.001
PLT	200.5	0.642	0.389	0.859	0.569 ~ 0.714	<0.001
D-dimer	1.06	0.624	0.701	0.567	0.556 ~ 0.693	0.001
FIB	4.14	0.641	0.667	0.587	0.573 ~ 0.709	<0.001
SII	1225.8	0.663	0.433	0.860	0.592 ~ 0.734	<0.001
PRE_1	0.707	0.808	0.756	0.761	0.757 ~ 0.866	<0.001

**Fig. 4:**
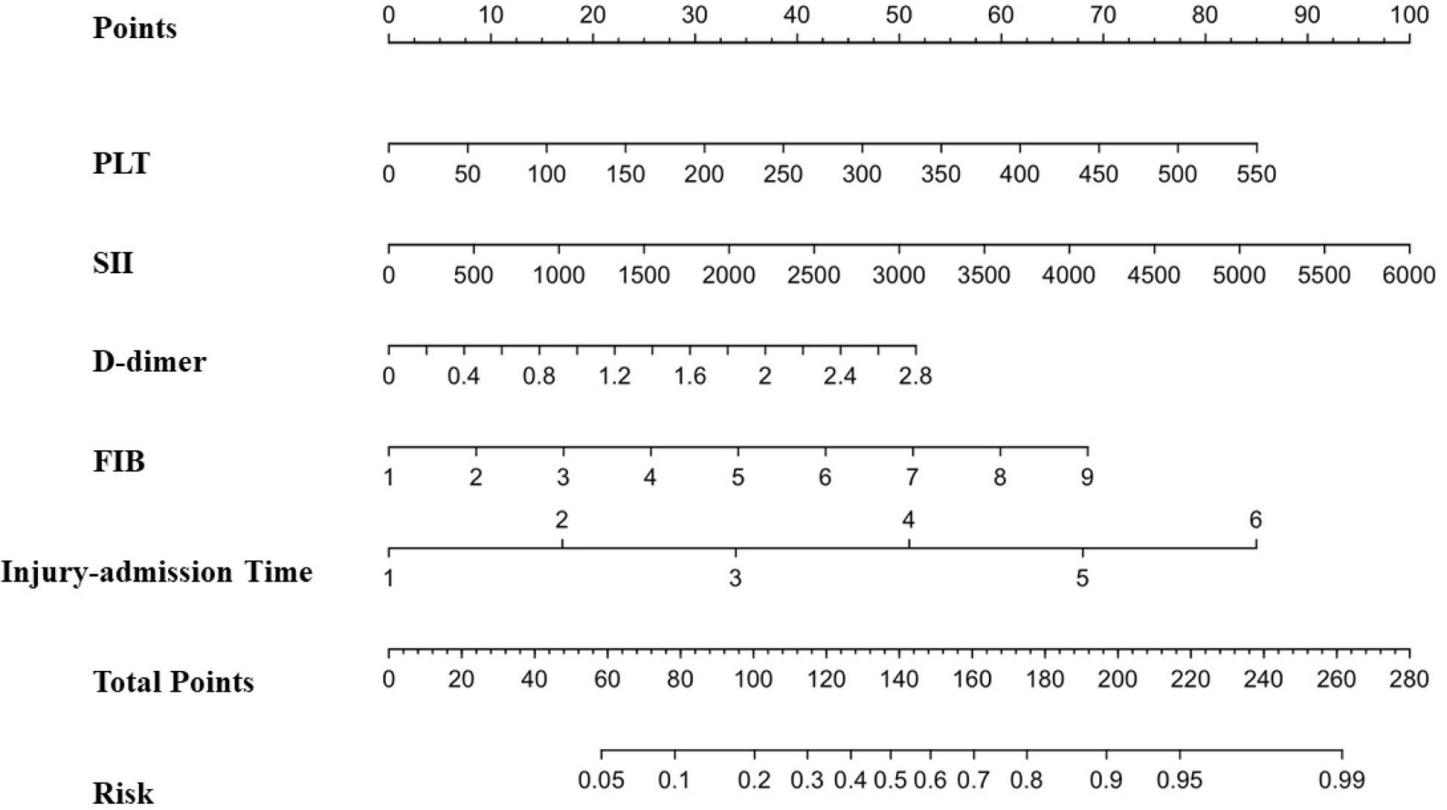
Construction of nomogram predictive model of preoperative DVT in elderly patients with hip fracture

**Fig. 5:**
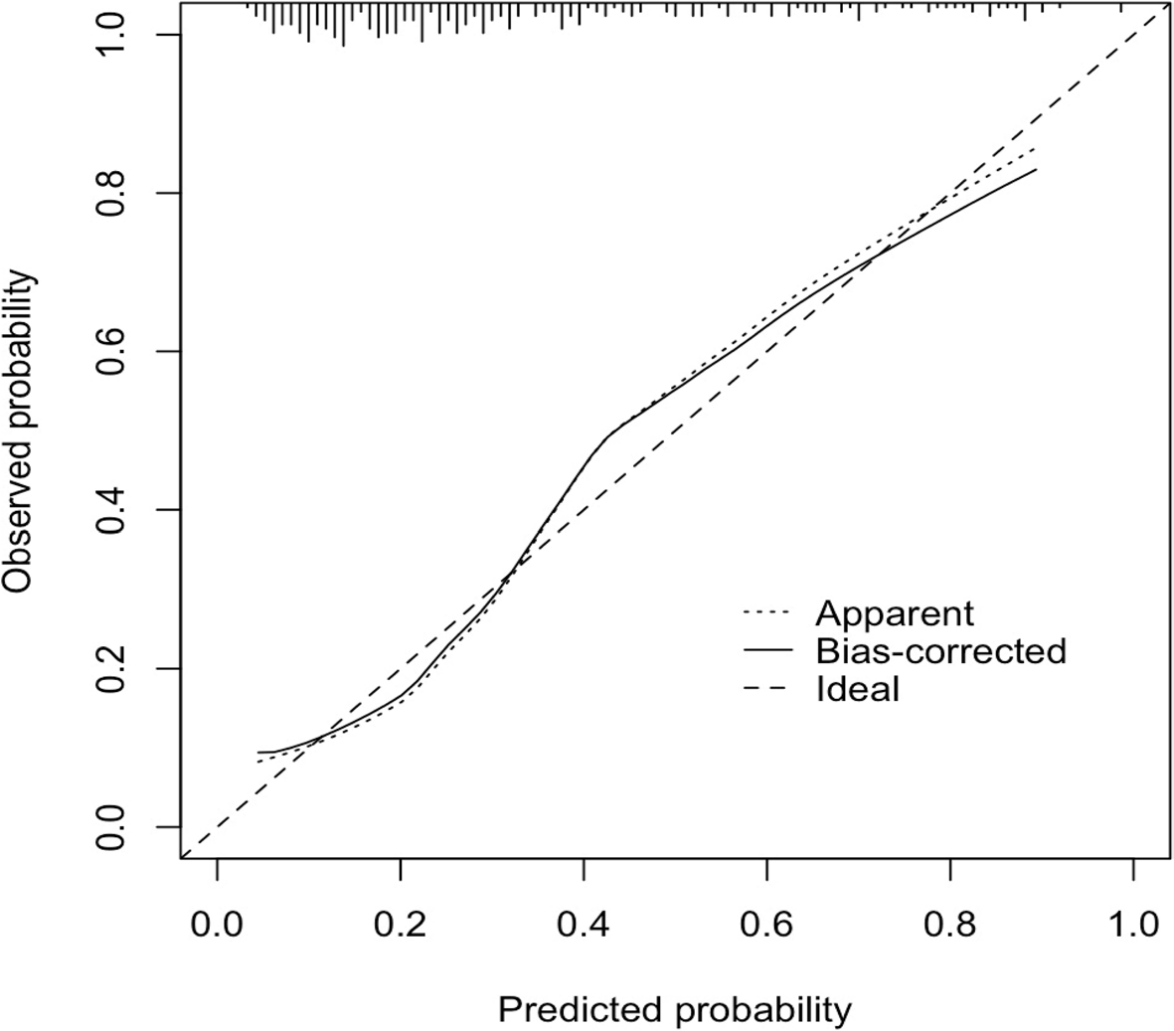
Calibration curve of the nomogram prediction model for preoperative DVT in elderly patients with hip fracture

Logistic regression analysis showed that factors including injury-admission time, PLT, D-dimer, FIB and SII were independent risk factors for DVT by analyzing the index admission data. The above risk factors were entered into the R “rms” package to construct a nomogram prediction model of DVT in elderly hip fracture patients with the use of R (Fig. 4). The scores of risk factors for DVT and the probability of DVT in elderly patients with hip fracture before surgery could be observed directly in the nomogram prediction model. Meanwhile, the construction of the nomogram prediction model, in which the C-index was 0.808 (95% CI = 0.757 ~ 0.866) (Fig. 5), needed to be verified to prove that the model had a good degree of fit, good predictive efficiency, high consistency between actual occurrence probability and prediction probability, and good accuracy for predicting DVT.

## Discussion

DVT is a common complication of hip fracture in older people. With the improvement of medical diagnoses, the prevalence of DVT has decreased, but the outlook is still not optimistic. Recent studies [18, 19] showed that 19.5–32% of hip fracture patients had preoperative DVT. In our study, the incidence of preoperative DVT in elderly patients with hip fracture was 32.8%, and the high incidence may be explained by the following: 1. the subjects of this study were elderly patients; 2. the injury site was the hip; and 3. most patients took a long time to transfer to a higher-level hospital or took a long time to follow up. Clinically, the length of preoperative preparation and the total length of hospitalization were prolonged, resulting in a higher incidence of DVT as the appointment for ultrasound examination was scheduled too far out [20]. In the results of our study, it was clear that the time from injury to hospitalization, PLT count, D-dimer level, FIB level, and SII were independent risk factors for hip fracture complicated with DVT in elderly patients, and SII could be considered a novel risk factor for predicting DVT. Although the risk value of the SII was small according to multifactor analysis, it had certain predictive efficiency and significance for the prediction of DVT. This study attempted to construct a nomogram prediction model by combining 5 independent risk factors to make a rapid, precise and effective prediction of preoperative DVT in elderly patients with hip fracture.

The delayed surgery of fracture patients resulting from a prolonged time from injury to hospitalization is one of the important factors contributing to the high incidence of DVT before surgery [21, 22]. Patients with hip fracture should be operated on as soon as possible within 48 hours to reduce the incidence of DVT [23]. Approximately 66% of patients with hip fractures delay surgery due to prolonged injury to hospitalization time for various reasons [24]. Usually, many patients are transferred from other hospitals to higher-level hospitals due to their older age, serious illness or even limited medical conditions. In particular, at a tertiary trauma center, most patients are admitted to the hospital after a few days of their injury. Meanwhile, patients with medical diseases often need further examinations, adequate preoperative preparation and screening for DVT, and so on after admission, which will delay the operation again, so it is particularly important to clarify the DVT situation immediately after admission. However, there is a conflict between adequate preoperative preparation and early operation, and achieving a comprehensive and scientific evaluation of patients is the key to avoiding unnecessary delays in surgery. In conclusion, a prolonged injury-admission time has a great impact on the DVT prevalence in elderly hip fracture patients. In this study, injury to hospitalization time was one of the independent risk factors for DVT (*P* < 0.001) through multivariate logistic regression analysis, for which the AUC = 0.682 (95% CI: 0.615 ~ 0.750), cutoff value was 3.5 days, OR = 1.881 (95% CI: 1.470 ~ 2.405), and sensitivity and specificity were 35.6 and 90.2%, respectively. This result was similar to the study of preoperative complicated DVT after elderly hip fracture, in which the article also suggested that patients with injury-admission time > 3 days should be treated as a high-risk group and preferentially selected for lower extremity vascular ultrasound with color Doppler [25]. In this study, the injury-admission time had a strong correlation and a good predictive efficiency with preoperative DVT in elderly patients with hip fracture, but the specificity of this factor was high, and the sensitivity was low.

Complicated DVT before surgery in elderly patients with hip fracture was associated with PLT count, D-dimer level, and FIB level in the comparison between the groups and the logistic regression analysis. The stress reaction of traumatic fracture could increase the breakdown of PLTs, while the activation of PLTs has great value in monitoring DVT. Niu [25] found that the risk was 2.02 times greater in elderly patients with hip fracture with a preoperative PLT count> 220 × 10^9^/L compared to patients without DVT. Another study [26] also found that elderly patients with femoral fractures had a 2.55-fold risk of developing DVT before operation when PLT count> 217 × 10^9^/L. In this study, multiple logistic regression analysis showed that PLT count was one of the independent risk factors for DVT (*P* < 0.05), with an AUC = 0.642 (95% CI: 0.569 ~ 0.714), cutoff value of 200.5 × 10^9^/L, OR = 1.008 (95% CI: 1.002 ~ 1.015), and sensitivity and specificity of 38.9 and 85.9%, respectively. The cutoff value of PLT count was similar to recent studies and had a great predictive efficiency, but this factor had high specificity, low sensitivity, and small risk values. In addition to the increase in PLT level, D-dimer level may also increase after fracture. Predicting the critical value of D-dimer in DVT is controversial. A study [27] showed that a preoperative D-dimer value > 4.01 mg/L was considered to be an important predictor of preoperative DVT in patients with traumatic fracture (*P* < 0.05), with a preoperative D-dimer AUC of 0.593 (95% CI: 0.533 ~ 0.652), a sensitivity of 71.2%, and a specificity of 44.83%. Another study stated that the sensitivity and specificity of the D-dimer level suggesting DVT were 71.4 and 78.6%, respectively, when the D-dimer level was>2.79 mg/L. [22] The optimal critical value of D-dimer was 1.0 mg/L, approximately twice the upper limit of the standard (0.5 mg/L) in patients with femoral fractures over 65 years old in a recent prospective study [26], and the risk of preoperative DVT increased by 3.5 when the D-dimer level was >1.0 mg/L. Therefore, appropriate adjustment of the cutoff value is necessary and valuable for the clinical judgment of perioperative complications of DVT in patients of different ages. Age-adjusted D-dimer values have been widely used in the prediction of DVT [28]. Many studies have focused on the use of early detection methods to predict the occurrence of DVT, especially blood D-dimer levels. However, the increase in the D-dimer level was also related to the degree of trauma, the degree of inflammatory reaction, age and other factors [29]. In this study, all the subjects were over 60 years old and included in the multivariate analysis. Logistic regression analysis showed that D-dimer level was one of the independent risk factors for DVT (*P* < 0.05). The sensitivity and specificity of predicting DVT in elderly patients with hip fracture were 56.7 and 70.1%, respectively, with an AUC = 0.624 (95% CI: 0.556–0.693) when the D-dimer level was > 1.06 mg/L (*P* < 0.05). The difference in the D-dimer value may be related to the different study subjects and the regions of the body being studied, which are affected by many factors, so interference is considerable. Relying only on the D-dimer value to screen patients with traumatic fractures for perioperative DVT is not enough [30]. In addition, monitoring FIB levels has clinical significance for predicting DVT. Wang [31] identified FIB level as one of the independent risk factors for DVT in their study of postoperative DVT due to thoracolumbar fracture caused by high-energy injury and pointed out that the cutoff value of FIB was 5.1 g/L, OR = 1.575, AUC = 0.580 (*P* < 0.05). Cheng [32] found that the combination of the three indices PAI-1, FIB and D-dimer had a very good predictive value for postoperative DVT in lower limb fracture by the analysis of ROC curves for the above three factors. In this study, multivariate logistic regression analysis showed that FIB level was one of the independent risk factors for DVT, with an AUC = 0.641 (95% CI: 0.573 ~ 0.709), cutoff value of 4.14 g/L, OR = 1.391 (95% CI: 1.094 ~ 1.768), and sensitivity and specificity of 66.7 and 58.7%, respectively. Compared with PLT and D-dimer, FIB level also has a great predictive efficiency, and the sensitivity of FIB is higher than that of PLT count and lower than that of D-dimer level, but the specificity is not high.

Studies have shown that the factors affecting DVT are not limited to the coagulation system but are also closely related to the immune inflammatory system. Saghazadeh [33] described several immune and inflammatory system components (cytokines, chemokines and various leukocyte subtypes) that are involved in the potential inflammatory process of DVT in a review. Branchford [10] also pointed out a possible mechanism by which an inflammatory reaction of the blood vessel wall leads to DVT, although the inflammatory system and the coagulation system together through a common pathway can lead to the activation of endothelial cells, PLTs and WBCs, therefore causing an inflammatory reaction, forming particles and inducing tissue factor (TF) to trigger the coagulation system. This article showed that the key to the formation of DVT is probably venous wall inflammation, but the role of specific immune regulation has not been clarified. Although the triad of Virchow greatly promotes our understanding of DVT, there is more evidence showing that the activation of the immune inflammatory system plays an important role in the pathophysiology of DVT [34]. The formation and dissolution of DVT are accompanied by a series of inflammatory and immune responses [35]. Alexandru [36] found that some peripheral blood-derived indices (NLR, PLR) changed significantly after fracture. In addition, researchers found that there was a significant correlation between the MLR, PLR, or NLR and fractures complicated by DVT [14, 16, 37]. However, the role of peripheral blood-derived indices in DVT is not always significant [38]. Recently, a new type of immune inflammation index, the SII, was constructed, which was different from the NLR, PLR and MLR. It is better and combines neutrophils, PLTs and lymphocytes and has a stronger predictive ability for some diseases. A recent study on the role of the SII in the formation of cerebral venous sinus thrombosis [39] reported that the SII was a potential index of poor prognosis in patients with acute/subacute cerebral venous sinus thrombosis. The predictive value of the SII (AUC = 0.792, *P* < 0.05) was higher than those of the NLR (AUC = 0.743) and PLR (AUC = 0.760) when the cutoff value of the SII was 1525.04 × 10^9^/L. Peng [40] found that the SII had a good ability to predict the preoperative production of DVT in elderly patients with hip fracture in their study, while ROC analysis showed that the AUC = 0.795 (95% CI: 0.71 ~ 0.88), and sensitivity and specificity were 53.8 and 92.3%, respectively, when the critical value of the SII was 847.78 × 10^9^/L. In this study, multivariate logistic regression analysis showed that the SII was one of the independent risk factors for DVT (*P* < 0.05), with an AUC = 0.641 (95% CI: 0.592 ~ 0.734) (*P* < 0.001), cutoff value of 1225.8 × 10^9^/L, OR = 1.002 (95% CI: 1.000 ~ 1.004), and sensitivity and specificity of 43.3 and 86%, respectively. The risk value of the SII was very small, but its specificity was very high, which indicated that inflammation and immune reactions had specific effects on DVT, resulting in the SII making up for the shortcomings of the low specificity of D-dimer level and introducing a new factor for clinical prediction of DVT.

In recent clinical research and practice, the Caprini thrombus risk score scale has been widely used, recognized, and recommended by many scholars and has a certain value for the evaluation of DVT [41]. However, in this study, the Caprini thrombus risk score of the admission data was not statistically significant in elderly patients with hip fracture, which was inconsistent with previous studies showing that the Caprini thrombus risk score could predict DVT [42]. There was no significant difference between the thrombus group (8.97 ± 1.00) and the nonthrombus group (8.72 ± 1.24) after analyzing the results of this study (*P* > 0.05), and the elderly patients with hip fracture and scores >5 points were all in the extremely high-risk group. There is a certain bias in this study because the number of patients may be small and some artificial subjective factors may be mixed in the rating scale, which may have a certain impact on the results. Moreover, the subjects were all high-risk elderly individuals, so there was no statistical significance between the groups. In addition, this study also confirmed that immune inflammatory indices, mostly WBC, NC, and MC levels, and the NLR, PLR and MLR, related to DVT after hip fracture in elderly patients were risk factors for DVT and were statistically significant compared to the nonthrombus group, but it was found that these factors could not perfectly predict the occurrence of DVT after hip fracture in older people through multivariate logistic analysis. PRE_1 (AUC = 0.808) was constructed by the analysis of the ROC curve combining five factors: time from injury to admission, PLT count, D-dimer level, FIB level and the SII. The sensitivity and specificity were 75.6 and 76.1%, respectively. PRE_1 had better predictive efficiency for DVT than any other single factor. In addition, a nomogram prediction model for elderly patients with hip fracture complicated with DVT was constructed, and five independent risk factors were scored directly so that the risk of preoperative DVT in elderly patients with hip fracture could be predicted more accurately, quickly and effectively.

The limitations of this study are as follows: First, this is a single-center, retrospective case–control study with a small sample size, which is prone to confounding factors and cannot provide a high level of evidence. The distribution of risk factors, such as age and sex, of the Caprini score was similar among the groups. No significant differences could be found based on the current sample size. Second, all patients were examined with color Doppler ultrasound after admission, but the delay in color Doppler ultrasound examination time in some patients may have caused the formation of preoperative DVT. Third, only the calibration map and c-index were used for the prediction of the constructed risk model to evaluate the accuracy of the model without any external validation methods, so sufficient data are needed to validate and supplement the model in the future. Fourth, the simple hip fractures in this study mainly included femoral neck fractures or intertrochanteric fractures, and this study has many other exclusion criteria (as pathologic fracture, thrombotic disorder or use of anticoagulant drugs, some DVT-related disease, and delayed admission time), resulting in a larger excluded sample size and the model use limited to simple hip fracture. In this experiment, 117 cases were excluded, such as those with multiple fractures. This study analyzed the basic data of patients admitted to the hospital, hematology indices, peripheral blood-derived indices and other factors to jointly predict the risk of DVT in elderly patients with hip fractures, so that high-risk patients could be screened out and intervention carried out as soon as possible to shorten the perioperative time in order to reduce the incidence of preoperative DVT of hip fractures in elderly individuals.

## Conclusions

Injury-admission time, PLT count, D-dimer level, FIB level, SII and other factors can be used as risk factors for predicting preoperative DVT of hip fractures in elderly individuals. A nomogram prediction model was constructed by combining 5 independent risk factors, including time from injury to admission PLT count, D-dimer level, FIB level, and SII, which had high predictive value for fractures complicated with DVT. This model use is limited to simple hip fracture.

## Data Availability

The datasets generated and/or analysed during the current study are not publicly available due to limitations of ethical approval involving the patient data and anonymity but are available from the corresponding author on reasonable request.

## Abbreviations

DVT: Deep venous thrombosis
PRE_1: Prediction probability_1
ROC: Receiver operating characteristic curve
AUC: Area under the curve
Index: Concordance Index
TF: Tissue factor
PLT: Platelet
FIB: Fibrinogen
SII: Systemic immune-inflammatory index
PE: Pulmonary embolism
MLR: Monocyte/lymphocyte ratio
NLR: neutrophi/lymphocyte ratio
PLR: Platelet/lymphocyte ratio
WBC: White blood cell
NC: Neutrophil count
MC: Monocyte count
LC: Lymphocyte count
PT: Prothrombin time
APTT: Activated partial thromboplastin time
TT: Thrombin time
CRP: C-reactive protein
ESR: Erythrocyte sedimentation rate
